# CD79A work as a potential target for the prognosis of patients with OSCC: analysis of immune cell infiltration in oral squamous cell carcinoma based on the CIBERSORTx deconvolution algorithm

**DOI:** 10.1186/s12903-023-02936-w

**Published:** 2023-06-21

**Authors:** Shucong Yao, Zixian Huang, Changji Wei, Yuepeng Wang, Hongwei Xiao, Shisheng Chen, Zhiquan Huang

**Affiliations:** 1 Department of Oral and Maxillofacial Surgery, Second Affiliated Hospital of Shantou University Medical College, 69 Dongxia North Road, Shantou, 515000 Guangdong China; 2 Nanhai Translational Innovation Center of Precision Immunology, Sun Yat-Sen Memorial Hospital, Guangzhou, China; 3 Department of Oral and Maxillofacial Surgery, Sun Yat-sen Memorial Hospital, Sun Yat-sen University, Guangzhou, Guangdong China

**Keywords:** Oral squamous cell carcinoma, Tumor-infiltrating immune cells, CD79A, Tumor microenvironment, Infiltration abundance, Target prediction

## Abstract

**Objective:**

To analyze the abundance of infiltrating tumor immune cells in patients with oral squamous cell carcinoma (OSCC) and to search for potential targets that can predict patient prognosis.

**Methods:**

A total of 400 samples from 210 patients with OSCC were collected using The Cancer Genome Atlas (TCGA) database. CIBERSORTx was used to evaluate the infiltration abundance of tumor immune cells. Potential target genes were searched to predict patient prognosis through case grouping, differential analysis, and enrichment analysis. Surgical excisional tissue sections of patients with oral squamous cell carcinoma admitted to the Department of Oral and Maxillofacial Surgery, Second Affiliated Hospital of Shantou University Medical College, from 2015 to 2018 were collected and followed up.

**Results:**

The CIBERSORTx deconvolution algorithm was used to analyze the infiltration abundance of immune cells in the samples. Cases with a high infiltration abundance of naive and memory B lymphocytes improved the prognosis of OSCC patients. The prognosis of patients with low CD79A expression was significantly better than that of patients with high CD79A expression.

**Conclusion:**

CD79A can predict the infiltration abundance of B lymphocytes in the tumor microenvironment of patients with OSCC. CD79A is a potential target for predicting the prognosis of patients with OSCC. This study provides novel ideas for the treatment of OSCC and for predicting patient prognosis.

## Background

Head and neck squamous cell carcinoma (HNSCC) is one of the most prevalent cancers worldwide [1]. As an important component of HNSCC, oral squamous cell carcinoma (OSCC) keeping with high morbidity and mortality [2]. Local recurrence, cervical lymph node metastasis, and distant metastasis have resulted in a poor survival rate of OSCC [3]. Research has revealed that the tumor microenvironment (TME) plays an important role in tumor immune cell infiltration in tumor spread, recurrence, metastasis, and immunotherapy response [4]. Tumor microenvironment coordinates adaptive immunosuppression of tumor cells through cytokines that maintain inflammatory characteristics [5], avoids immune destruction, and is conducive to tumor progression, angiogenesis, cell invasion and metastasis [6–8]. By eliminating pathogens, immune cells play an important auxiliary role in maintaining the integrity and function of tissues in the process of homeostasis, infection, and noninfectious disturbances, which affect the clinical outcome of tumors. Moreover, the TME is constantly changing as the tumor progresses, which poses a great challenge to the treatment of malignant tumors [9].

The influence of tumor immune cells on the occurrence and development of HNSCC has not been fully elucidated, and how these cells might affect patient prognosis is not yet clear. In recent years, an increasing number of radiotherapy and chemotherapy methods have been used in the treatment of HNSCC, but the five-year survival rate of many patients with HNSCC is still low [10].

With scientific and technological advances, especially next-generation sequencing technology, rich and diverse databases have enabled us to observe the heterogeneity of tumors at the genetic level [11].

Therefore, to investigate the impact of the TME on the occurrence, development, metastasis, and chemotherapy resistance of HNSCC and thus on patient prognosis, we accessed 400 OSCC samples from HNSCC samples of The Cancer Genome Atlas (TCGA) database and used CIBERSORT to analyze the infiltration abundance of 22 types of immune cells in these samples. Combined with clinical information, this study aimed to find clues for further exploration and potential targets that can predict patient prognosis.

## Methods

### Sample database and screening

As of April 1, 2022, the data of 400 OSCC samples from HNSCC samples were downloaded from The Cancer Genome Atlas Program (TCGA, https://portal.gdc.cancer.gov/) for further analysis. These samples were from 210 cases of OSCC from the group of HNSCC samples, which included squamous cell carcinoma tissues of the tongue, mouth floor, gingival, palate, oropharynx, and salivary gland.

### Evaluation of immune cell infiltration

We used the CIBERSORTx database to evaluate the infiltration abundance of tumor immune cells. The downloaded samples were analyzed using limma package [12], and the average gene expression levels of the repeats were calculated. The sample matrix was uploaded on the official website of CIBERSORTx (CIBERSORTx (stanford.edu)), and the proportion of immune cells in the sample was calculated. The samples with *P < 0.05* were filtered out. Finally, 225 samples were obtained, of which 14 samples were normal tissue samples and 211 samples were tumor samples.

### Case grouping and differential analysis

The obtained 225 samples were evaluated using the ConsensusClusterPlus package [13]. The most suitable classification method was found, and the samples were divided into four groups through the Delta map and the heatmap. The survival analysis of each group was performed by survival curves using the survminer package (https://github.com/kassambara/survminer).

We used the edgeR Package [14–16] to perform the gene differential expression analysis of clusters A and B. Genes with |log2FC| > 1 and *P < 0.05* were screened out.

### Enrichment analysis and survival prognosis analysis

Gene enrichment analysis was performed online through the Metascape website. (https://www.Metascape.com) to identify relevant genes, multivariate Cox regression was used to identify those significantly affected patient survival prognosis. Kaplan-Meier (K-M) method was used to verify the difference in survival prognosis of patients with different expression levels of selected genes by survminer package.

### Correlation between target genes and tumor immune cell infiltration

The TIMER2.0 (http://timer.cistrome.org/) database was used to examine the correlation between the screened genes and related immune cells to verify the correlation between the infiltration abundance and the expression of the screened genes.

### Obtaining clinical samples and follow-up data

From 2015 to 2018, a total of 117 patients with OSCC were treated in the Department of Oral and Maxillofacial Surgery, Second Affiliated Hospital of Shantou University Medical College, and the patients underwent surgical treatment. Intraoperative frozen sections were used to confirm that the surgical boundary was expanded to the normal range. After surgery, a pathological diagnosis was determined by the Department of Pathology, Second Affiliated Hospital of Shantou University Medical College. All clinical staging was based on the eighth edition of TNM staging for head and neck squamous carcinoma as specified by the American Joint Committee on Cancer (AJCC) and the Union for International Cancer Control (UICC). Patient follow-up information was collected through telephone contact.

### Immunohistochemistry

Immunohistochemical staining was performed according to standard protocols [17]. After deparaffinization, antigen retrieval was conducted using Tris-EDTA buffer (#C1038, Solarbio) in microwave oven for 15 min. Briefly, the tissue sections were blocked sequentially with 3% H_2_O_2_ and normal serum and then incubated with primary antibodies at 4 °C overnight. Tissues were incubated with a biotinylated secondary antibody and conjugated with a streptavidin-HRP complex (ready-to-use SP kit; Zhongshan Co., China). Finally, the sections were visualized with 3–3′-diaminobenzidine, counterstained with hematoxylin and mounted. The samples were rinsed with PBS between each step.

### Evaluation of immunohistochemistry

The tissues were evaluated by two pathologists who assessed the number of positive cells and staining intensity. Positive staining was assessed using a semiquantitative scoring system. The percentage of positive cells was scored as follows: 0 (no staining), 1 (< 1/3 staining), 2 (1/3 to 2/3staining), and 3 (> 2/3 staining). Staining intensity was scored as follows:0 (negative), 1 (weak positive), 2 (medium positive), and 3 (strong positive). The final evaluation of protein expression was based on the sum of the scores for percentage positivity and intensity. A score of 0–2 was defined as low expression, while a score > 2 was defined as high expression.

### Statistical analysis

All statistical analyses were performed using R software (v4.1.2). Clinical patient survival was analyzed using the Kaplan–Meier method in the survival package, and the significance of the difference between the survival curves was determined using the log-rank test. Multivariate Cox regression was used for the multivariate analysis of survival. Multivariate analysis of variance and the chi-square test were used for correlation analysis.

## Results

### Immune cell infiltration was abundant in HNSCC

**Fig. 1:**
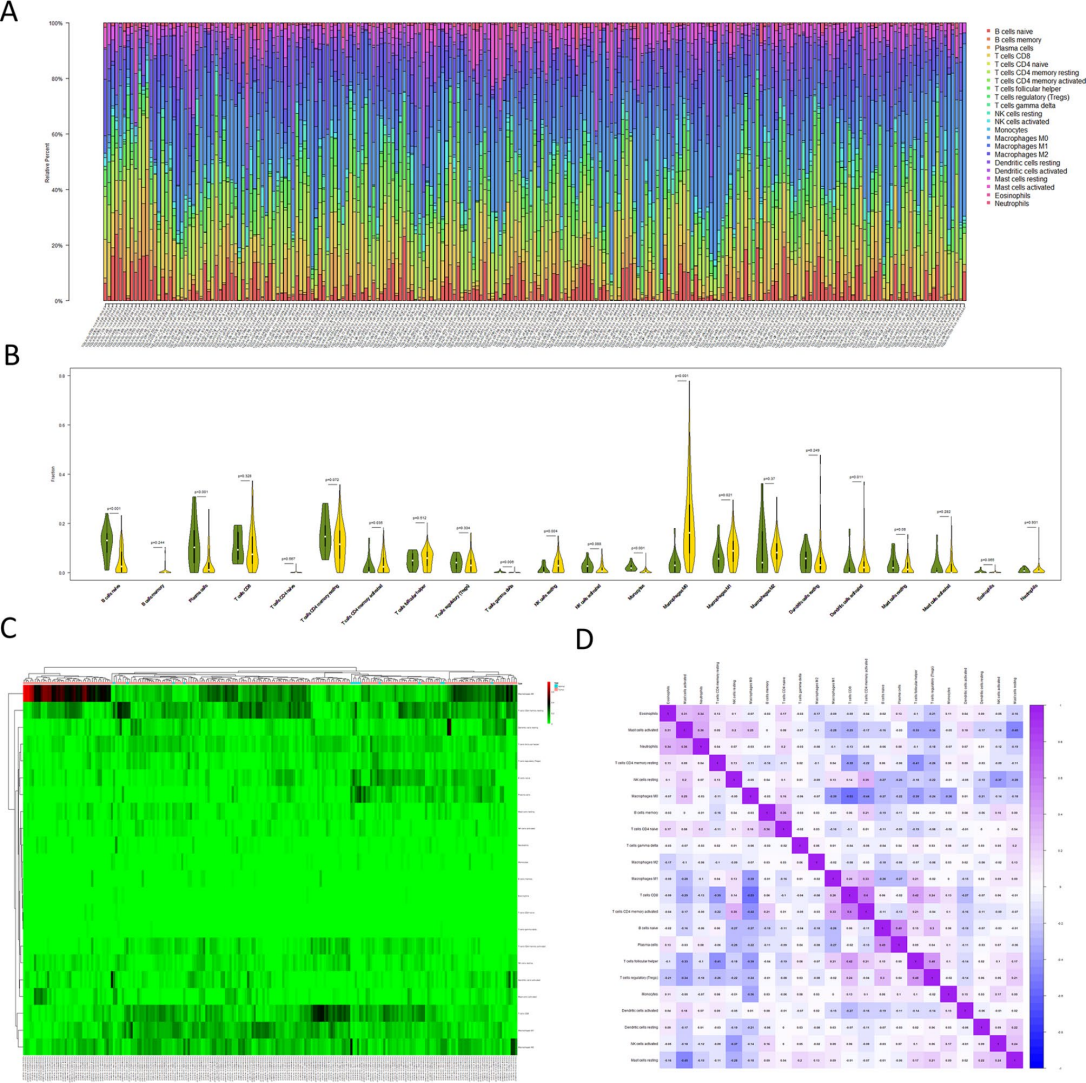
**Differences between normal tissue samples and tumor tissue samples** A: Bar graph showing the infiltration distribution of 22 types of immune cells in normal tissues and tumor tissues B: Violin graph showing the differences in the infiltration levels of 22 types of immune cells in normal tissues and tumor tissues C: Heatmap showing differences in the infiltration distribution of 22 types of immune cells in normal tissues and tumor tissues D:Correlation heatmap showing the correlations between the 22 types of immune cells

We performed tumor immune analysis and evaluation on the selected samples through the CIBERSORTx deconvolution algorithm. CIBERSORTx calculated and displayed the infiltration abundance of 22 types of immune cells in the samples. The results indicated that compared with normal samples, the infiltration abundance of immune cells in tumor samples was more obvious (Fig. 1A). At the same time, the infiltration abundance of naive B cells (*P < 0.001*), plasma cells (*P < 0.001*), and monocytes (*P < 0.001*) in normal samples were significantly higher than that in tumor samples. The infiltration abundance of the memory CD4 T cells (*P = 0.035*), resting NK cells (*P = 0.004*), M0 macrophages (*P < 0.01*), M1 macrophages (*P = 0.021*), and dendritic cells activated (*P = 0.011*) in tumor samples was significantly higher than that of the normal samples. The correlation heatmap indicates a positive correlation between memory CD8 and CD4 T cells, and a positive correlation between CD4 naïve T cells and memory B cells. And a negative correlation between M0 macrophages and both CD8 and CD4 T cells (Fig. 1A-D).

### B lymphocyte infiltration of tumors affected the patient survival rate

**Fig. 2:**
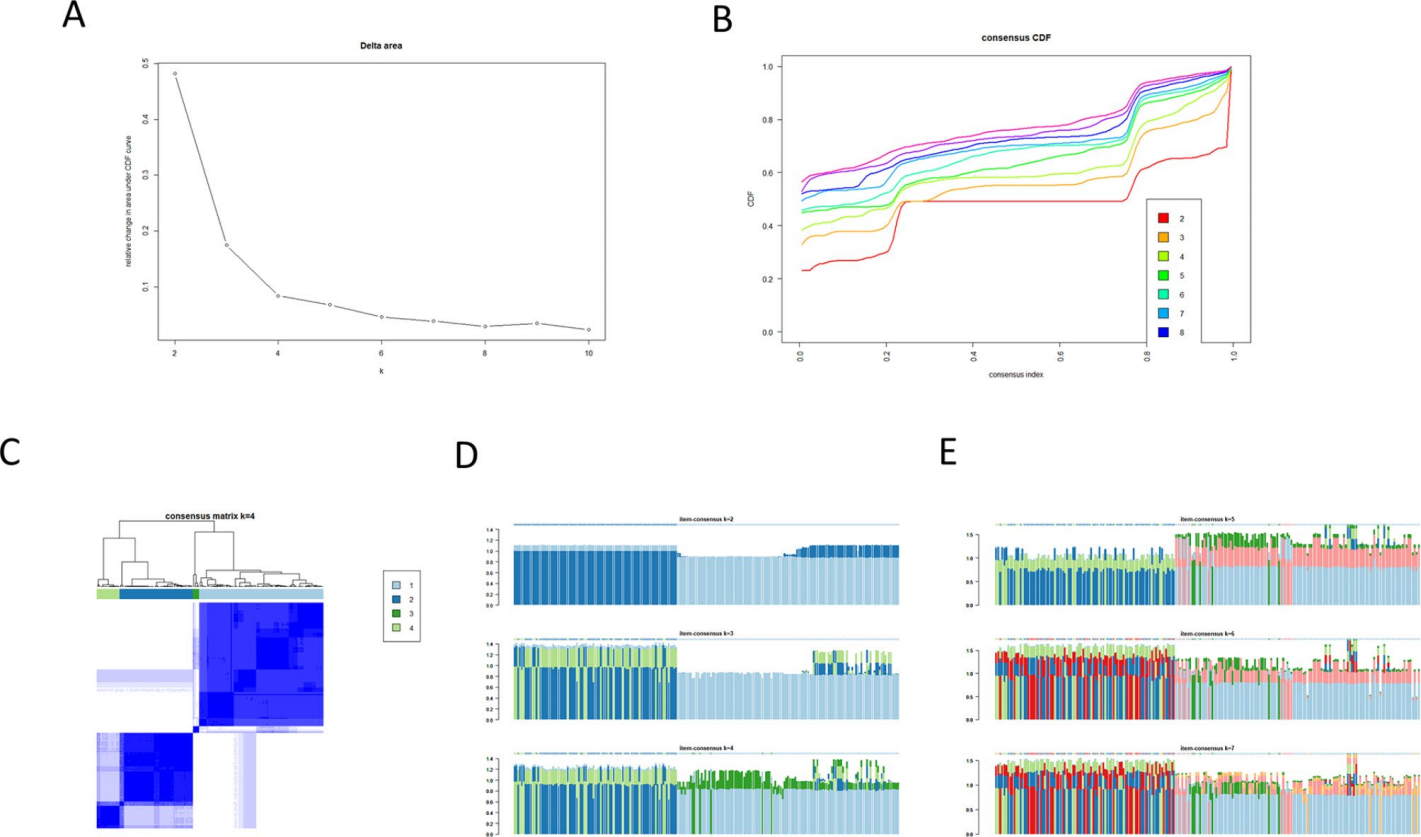
**Consensus cluster analysis using the ConsensusClusterPlus package** A: The delta plot shows that the area under the CDF curve no longer changed significantly after k = 4. B: CDF curve plot C: The samples were distributed into four groups by CM plot D&E: The IC plots indicated that k = 4 was the optimal number of classifications

We used the consensus clustering algorithm in the ConsensusClusterPlus package to perform consistent clustering analysis. Through the ConsensusClusterPlus package, we set reps = 1000, pItem = 0.8, and pfeature = 1. Using the delta plots, consensus matrix (CM) plots, item-consensus (IC) plots, and cumulative distribution function (CDF) plots, CC algorithm was visualized to facilitate the finding of the optimal number of classifications. The approximate maximum k value of the distribution reached under the condition of maximum stability was found through the CDF plots, and the relative change in area under the CDF plot curve was represented by delta plots. The results indicated that when the k value was 4 (Fig. 2A), the change in the area under the CDF curve was no longer significant. At this point, k = 4 was concluded to be the optimal number of classifications (Fig. 2B). Then, all cases were divided into four categories using the optimal method and represented by CM plots (Fig. 2C). Each colored rectangular bar in the IC diagram represents each sample, and the height of the rectangle corresponds to the IC value. The consistent clusters were marked with colored asterisks on the rectangle, which is convenient for viewing which sample heights represent one group and which samples are mixed with other groups. The less mixed the color of the color rectangle, the more consistent the group is. The IC plots reveal that when k is greater than or equal to 5, the color of each color bar becomes increasingly mixed; similarly, k = 4 is the optimal classification number (Fig. 2D-E). According to the results, we divided the samples into four groups and performed cluster analysis as the optimal plan.

**Fig. 3:**
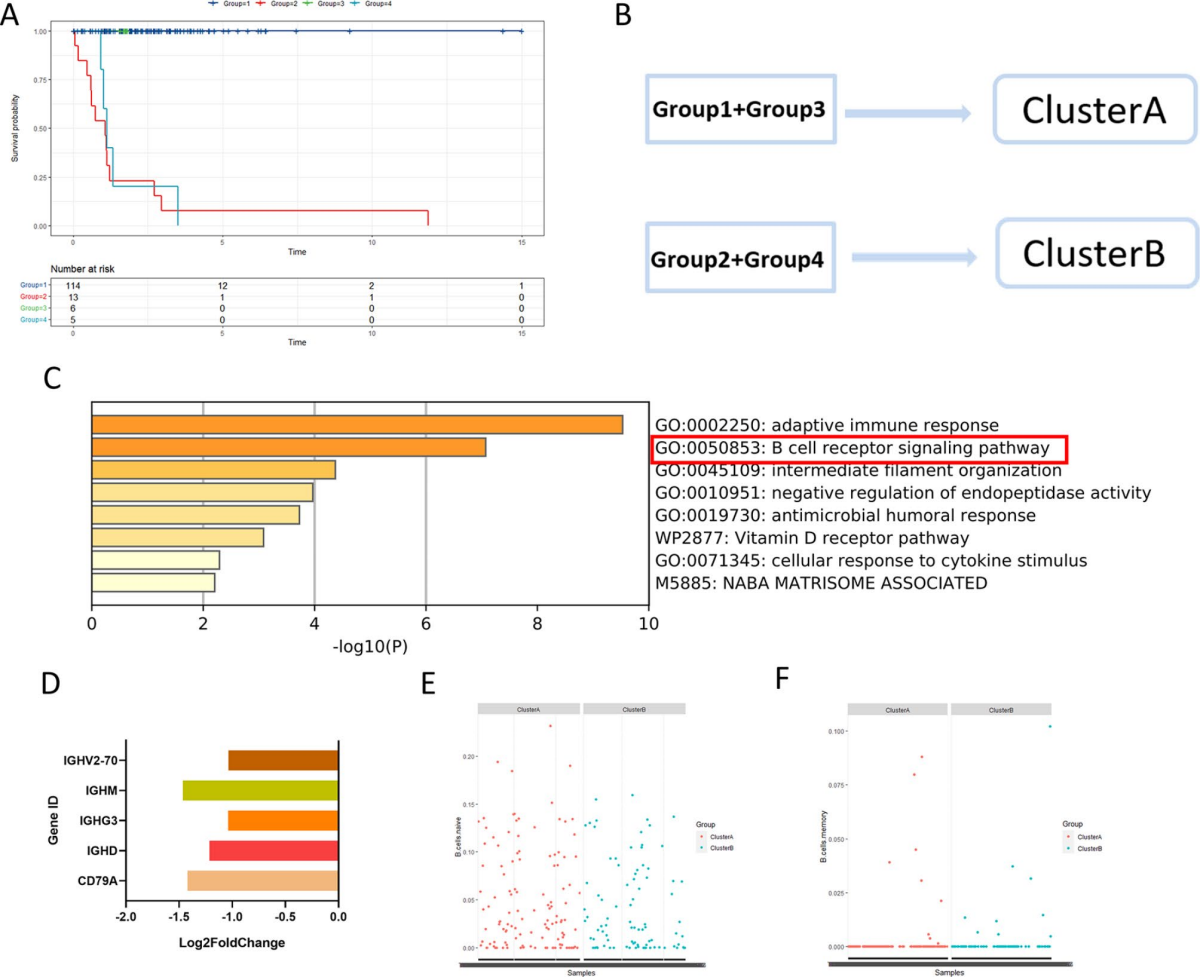
**Group analysis and Enrichment analysis** A: The Survminer package was used to perform K-M survival analysis, and it was found that the prognoses of the first and third groups were significantly better than those of the second and fourth groups B: The first and third groups were combined into cluster A; the second and third groups were combined into cluster B C: The enrichment analysis results of the Metascape database D: Five genes were associated with B-cell receptor signaling pathways, and all had significant differential expression E & F: the infiltration abundance of memory B cells and naive B cells were seemingly different between cluster A and cluster B

After analyzing the survival of these four groups using the Survminer package, we found that the survival prognoses of the first and third groups were significantly better than those of the second and fourth groups (Fig. 3A). To explore the reasons for the significant difference in survival prognosis, we classified the first and third groups as cluster A and the second and fourth groups as cluster B (Fig. 3B). Using the edge R package, we performed the differential analysis of the cluster A and cluster B gene expression levels, and 30 genes with significant differential expression between the two clusters were screened with |logFC| > 1 and *P < 0.05*.

### Enrichment analysis

After differential analysis, the 30 genes with significant differential expression were screened for online gene enrichment analysis using the Metascape website. Five genes, CD79A, IGHD, IGHG3, IGHM, and IGHV2-70, were enriched in the B-cell receptor signaling pathway (Fig. 3C, D; Table 1). We also note that the infiltration abundance of memory B cells and naive B cells were seemingly different between cluster A and cluster B. (Fig. 3E, F)

**Table 1:** Five genes with significant differential expression enriched in the B-cell receptor signaling pathway

Gene ID	Log2FoldChange	Log2CPM	P value	FDR
CD79A	-1.420365627	4.929473725	0.00000209	0.0007769
IGHD	-1.214312563	5.298169419	0.001075435	0.042693127
IGHG3	-1.038873442	8.403678554	0.000975217	0.04112342
IGHM	-1.464552247	8.375177082	1.23E-05	0.002856521
IGHV2-70	-1.03617177	5.143032756	0.004168784	0.083538477

### Survival prognosis analysis

**Fig. 4:**
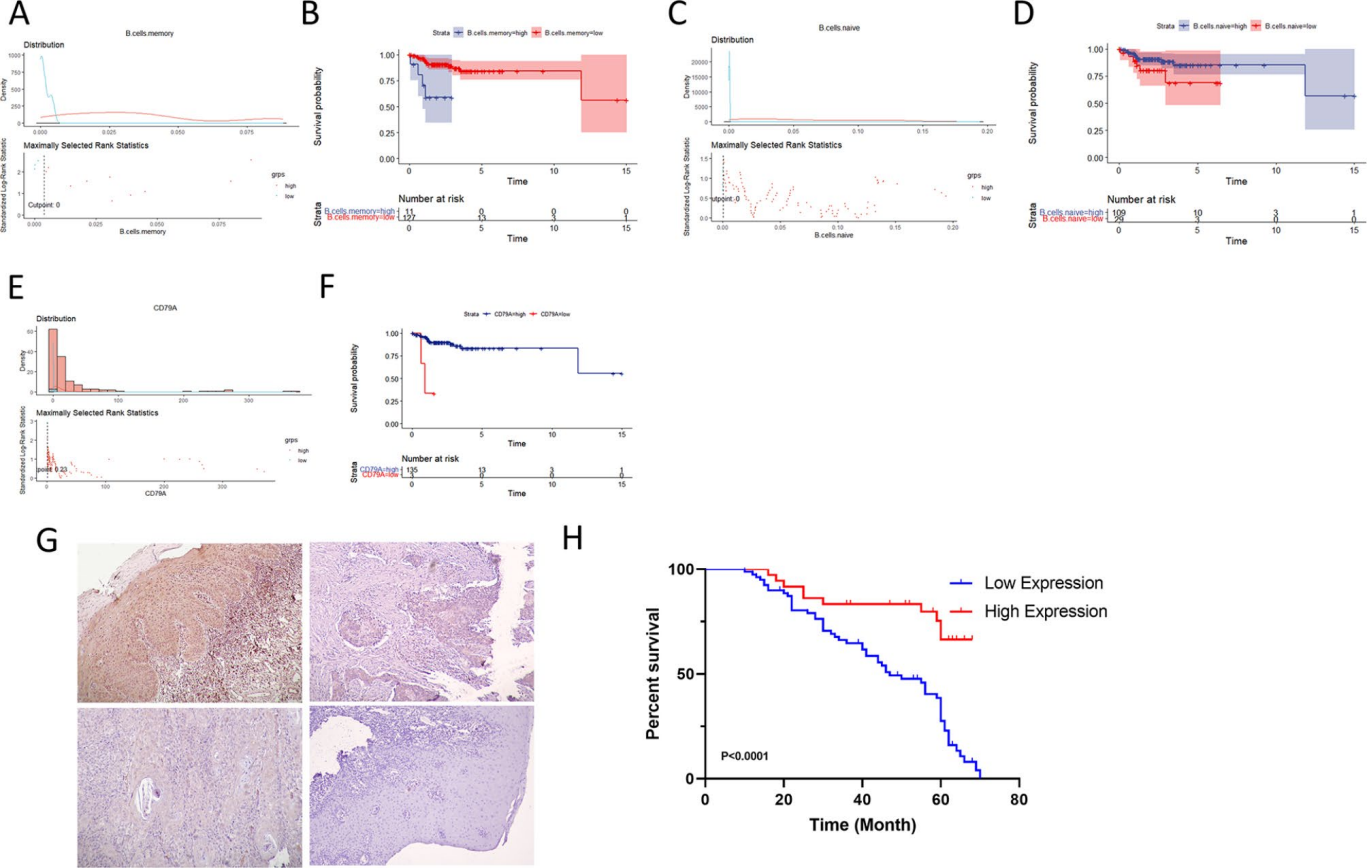
**Survival analysis based on the infiltration abundance of B lymphocytes** A&C: According to the infiltration abundance of naive B cells and memory B cells, patients were divided into high- and low-infiltration abundance groups using the surv_cutpoint function B&D: Patients with different B lymphocyte infiltration abundances showed different survival rates E: The surv_cutpoint function was used to divide the patients into high- and low-expression groups according to the expression levels of CD79A. F: The survminer package was used for survival analysis according to the different expression levels of CD79A G: Representative images of CD79A expression in surgically resected tissues of patients with oral squamous cell carcinoma in immunohistochemistry experiments H: Follow-up data indicated that the prognosis of patients with high CD79A expression was significantly worse than that of patients with low CD79A expression (*P < 0.0001*)

Through the “Surv_cutpoint” function in survminer package, an optimal cut point of the infiltration abundance of naive B cells and the memory B-cell infiltration in all samples were selected: 0.000621206 for naive B cells and 0.003937039 for memory B cells (Fig. 4A, C). According to the cut points, the infiltration abundance of cells was divided into high and low abundance, and K-M survival analysis was performed based on the results. The results indicated that patients with high naive B cell infiltration abundance had a better prognosis than those with low infiltration abundance, while patients with a high infiltration abundance of memory B cells had a poorer prognosis than those with low infiltration abundance (Fig. 4B, D).

Similarly, optimal cut points for the gene expression abundance of the five genes enriched in the B-cell receptor signaling pathway of each sample were selected using the “surv_cutpoint” function of the survminer package to create a division between high and low gene expression. Through the forward/backward multivariate Cox regression analysis, we found among the five genes enriched in the B-cell receptor signaling pathway, low expression of CD79A may potently affected the survival prognosis of patients (Table 2). The K-M survival analysis was performed based on the high and low gene expression levels. The results indicated that the prognosis of patients with low expression of CD79A was lower than that of patients with high expression of CD79A (Fig. 4E, F).

**Table 2:** Result of multivariate Cox regression

	coef	exp(coef)	se(coef)			z Pr(>|z|)	
CD79Alow	1.6202	5.0541	0.3797	0.842		0.0455*	
IGHMlow	0.9601	2.6119	0.5858	1.639		0.1012	
Signif. codes: 0 ‘***’0.001 ‘**’0.01 ‘*’0.05 ‘.’0.1 ‘ ’1							
	exp(coef)	exp(-coef)	lower 0.95		upper 0.95		
CD79Alow	5.054	0.1979	0.9012		28.345		
IGHMlow	2.612	0.3829	0.8286		8.233		

Concordance0.615(se = 0.062 )

Likelihood ratio test = 7.9 on 2 dfp = 0.02

Wald test = 11.64 on 2 dfp = 0.003

Score (logrank) test = 17.64 on 2 dfp = 1e-04

### Clinical samples and follow-up information indicated that CD79A affected the survival prognosis of patients

From 2015 to 2018, a total of 117 patients with oral squamous cell carcinoma were admitted and treated in the Department of Oral and Maxillofacial Surgery, Second Affiliated Hospital of Shantou University Medical College, and underwent surgical treatment. Intraoperative frozen sections were used to confirm that the surgical boundary was expanded to the normal range. Immunohistochemistry of the pathological tissues after resection revealed that CD79A could be expressed to different degrees in oral squamous cell carcinoma tissues (Fig. 4G). According to the follow-up data analysis, the survival prognosis of patients with high CD79A expression was significantly better than that of patients with low CD79A expression (*P < 0.0001*) (Fig. 4H). Survival was not significantly correlated with the patient’s sex, age, primary tumor location, clinical stage or degree of tumor differentiation but significantly correlated with lymph node metastasis (*P = 0.011*) (Table 3). Although the result showed that the clinical stage has no statistical differences with the expression level of CD79A, we still found out that more patients with low CD79A expression were in the stage III (n = 63) compared with patients with high CD79A expression.

**Table 3:** Clinical correlation analysis of CD79A and oral squamous cell carcinoma

		-	+	p value
Age	Mean ± SD	57.68 ± 11.30	57.50 ± 11.13	0.4948
Sex	Male	48	19	
	Female	32	18	0.9837
T staging	1	2	7	
	2	7	19	
	3	63	9	
	4	8	2	0.0542
Degree of differentiation	High	31	14	
	Medium	27	11	
	Low	22	12	0.5434
Lymph node metastasis	Yes	68	6	
	None	12	31	0.011
Primary lesion location	Lingual margin	31	15	
	Dorsum	5	7	
	Gingiva	11	2	
	Mouth floor	9	7	
	Cheek	16	5	
	Palate	3	0	
	Lip	2	0	
	Oropharynx	3	1	0.9373

In summary, we believe that CD79A expression not only affects the infiltration abundance of B lymphocytes in tumor tissues but can also be used as a potential target to analyze and predict the clinical prognosis of patients.

## Discussion

HNSCC is currently one of the most prevalent cancers worldwide [2], with a high mortality rate, recurrence rate, and metastasis rate [18]. As an important component of the head and neck squamous cell carcinoma, five-year survival rate of OSCC keeping below 50% [19]. In TME, tumor immune cell infiltration has been increasingly shown to affect patient treatment and prognosis. In different types of malignant tumors, infiltrations of various immune cells positively impact the survival and prognosis of patients [20]. In HNSCC, there are some new studies on tumor immune infiltration. Eberhardt et al. studied CD8 T cells from human HPV positive head and neck cancer patients and identified several epitopes from HPV E2, E5, and E6 proteins, and found that there are cellular mechanisms in HPV-positive head and neck cancer in response to PD-1 blocking [21]. Duhen et al. suggested that a higher frequency of CD103 CD39 tumor infiltration into CD8 T cells in patients with head and neck cancer predicted a better prognosis [22]. Chi et al. discovered 17 Natural killer cells signature and their nomograms demonstrate excellent prediction of prognosis in HNSCC patients. [23]. Not only to guide clinical treatment [24], the reasonable use of public databases and full mining of the information can provide effective guidance and design for basic experiments [25, 26].

Compared with T lymphocytes, there are relatively few studies on the function of B lymphocytes in the TME [27].

The traditional methods to identify immune cells in tissue samples are mostly immunohistochemistry or flow cytometry, but their disadvantages are clear. Immunohistochemistry requires multiple parameters to accurately divide the population and thus cannot identify many immune populations and does not perform well in capturing functional phenotypes. For flow cytometry, the samples must be processed within a short period after the samples are obtained, and its technical sensitivity is high; improper operations can easily cause errors, which may lead to the loss of fragile cell types and the distortion of gene expression profiles. CIBERSORT uses the deconvolution algorithm to obtain aggregated high-dimensional data from the cell mixture and infers the cell composition through calculation, which can better avoid these drawbacks [28, 29]. Although a variety of deconvolution algorithms have been used to infer the immune cell infiltration of tumor samples, CIBERSORT uses support vector regression to improve the performance of deconvolution algorithms through the combination of feature selection and robust mathematical optimization techniques [30]. In our study, we used CIBERSORT to calculate the infiltration abundance of 22 types of immune cells in HNSCC and attempted to identify a pattern.

In tumor tissues, B lymphocytes account for a high proportion of cells and are composed of activated, antigen-presenting, and memory B-cell subpopulations. These cells produce antibodies that directly target tumors, promote tumor recognition and tumor cell clearance through the activation of macrophages and complement cascades and antibody-mediated cytotoxicity, and play an important role in tumor antigen identification [31]. Sometimes B cells have a promoting effect on tumors, which is usually associated with the presence of immunosuppressive B-cell subsets called “regulatory B cells”, which may support tumor progression through various mechanisms. The existence of this antagonistic effect is due to the influence of different internal tumor environments, which cause the differentiation of B cells into different functional phenotypes and the different consequences of the immune response to different types of immunogenic tumor antigens [32]. In a variety of malignant tumors, it has been confirmed that the infiltration abundance of B lymphocytes significantly correlates with patient prognosis [32, 33].

As a signal transduction component of the pre-B-cell receptor, CD79A promotes the infiltration and recurrence of B-cell precursor acute lymphoblastic leukemia in the central nervous system [34]. CD79A is an important target of classical Hodgkin’s lymphoma; patients with high CD79A expression have a worse prognosis than those with low CD79A expression [35]. A mouse model confirmed that CD79A allows bone marrow-derived suppressor cells to maintain their immature state, enhances the ability to suppress T-cell proliferation, stimulates their migration, and induces the secretion of protumor cytokines such as IL-6 and CCL22, thereby promoting tumor growth [36].

At this point, our preliminary analysis indicated that CD79A expression in clinical patients significantly correlated with OSCC. Using the sample data collected from the TCGA database and the patient information, CIBERSORTx was used to analyze the differences in the infiltration abundance of immune cells in the samples. Combined with the prognosis in the patient information, we found that naive B cells and memory B cells in OSCC do affect patient prognosis. In addition, the high expression of CD79A suggests that the TME of patients with OSCC has a high infiltration abundance of B lymphocytes and is a potential target for predicting the prognosis of patients with OSCC. The clinical follow-up data and immunohistochemistry experiments revealed a significant correlation with the survival prognosis of patients, which provides a new direction for the treatment of OSCC.

## Conclusions

Our findings reveal that B lymphocyte plays an important role in the TME of OSCC. High expression of CD79A might be a potential influence target of the prognosis of patients with OSCC. The results also provide novel ideas for predicting patient prognosis. However, more basic experiments and clinical studies are still needed to evaluate the validity and reliability of this conclusion.

## Data Availability

Our datasets were derived from the following public domain resources: The Cancer Genome Atlas Program. (https://portal.gdc.cancer.gov/) The datasets used for the current study are available from the corresponding author on reasonable request. All data generated or analysed during this study are included in this published article.

## List of abbreviations

TCGA: The Cancer Genome Atlas database
NHSCC: head and neck squamous cell carcinoma
OSCC: oral squamous cell carcinoma
TME: tumor microenvironment
AJCC: American Joint Committee on Cancer
UICC: the Union for International Cancer Control
PBS: phosphate-buffered saline
CM: consensus matrix plots
IC: item-consensus plots
CDF: cumulative distribution function
